# Nephrin – a biomarker of early glomerular injury

**DOI:** 10.1186/2050-7771-2-21

**Published:** 2014-11-23

**Authors:** Yogavijayan Kandasamy, Roger Smith, Eugenie R Lumbers, Donna Rudd

**Affiliations:** Department of Neonatology, The Townsville Hospital, 100 Angus Smith Drive, Douglas, QLD 4814 Australia; Hunter Medical Research Institute, Mothers and Babies Research Centre, John Hunter Hospital, The University of Newcastle, Callaghan, NSW 2310 Australia; College of Public Health, Medical and Veterinary Sciences, The James Cook University, Townsville, QLD 4814 Australia

**Keywords:** Nephrin, Nephrinuria, Podocytopathy, Glomerular injury, Biomarker

## Abstract

Nephrin is a 180 KD trans-membrane protein expressed in glomerular podocytes. It was first identified in children with congenital nephrotic syndrome of the Finnish type (NPHS1). Nephrin forms an integral part of podocytes, which—together with endothelial cells and the basement—form the glomerular filtration barrier. Podocytopathies result in the detection of nephrin in the urine. We reviewed the literature to determine if urine nephrin measurements could become useful as a biomarker to detect early podocyte injury. Our search identified a total of 19 studies that have been published to date. The most common clinical conditions for which urine nephrin analyses were carried out included diabetic nephropathy, glomerulonephritis and pre-eclampsia. Nephrin measurement was carried out using commercially available ELISA kits, the messenger ribonucleic acid real-time polymerase chain Reaction, or electrophoresis. Nephrinuria showed positive correlation with proteinuria and severity of podocyte injury. In two studies, the level of nephrinuria declined in conjunction with clinical improvement in the patient following immunosuppressive treatment. Currently, there is no published data on the value of measuring urinary nephrin in pediatric patients.

## Introduction

Congenital nephrotic syndrome of the Finnish type (NPHS1) is an autosomal recessively inherited disorder that is characterized *in utero* by fetal hydrops. It also presents as nephrotic syndrome in neonates, a progressive disease that leads to massive proteinuria and death during the first two years [1]. This condition was first reported in the Finnish population [2], but it has subsequently been recognized to occur elsewhere. Specifically, by using linkage analysis in a cohort of 17 Finnish families, the genetic locus for this disorder was mapped to 19q12-q13 [3]. The NPHS1 (Nephrosis 1, congenital, Finnish type) gene product Nephrin was subsequently identified as a 180 KD transmembrane protein expressed in glomerular podocytes [1]. Nephrin has also been detected in the pancreas, brain, spinal cord and lymphoid tissues [4], although its role in these tissues has not been identified.

### Nephrinuria and podocytopathies

Nephrin forms an integral part of podocytes, which—together with endothelial cells and the basement—form the glomerular filtration barrier [5]. The filtration barrier consists of a basement membrane,a glomerular endothelial cell monolayer, and a podocyte monolayer on the urinary side [6]. Podocytes have four main functions, which all depend on their unique and specialized architecture. These functions are: the regulation of glomerular permeability selectivity, provision of structural support for the glomerular capillary, remodeling of the glomerular basement membrane (GBM), and endocytosis of filtered proteins [7, 8]. Podocytes can become injured in human and experimental glomerular diseases and conditions that cause podocyte injuries; these are collectively known as podocytopathies [7]. Pollak et al. proposed the concept of inherited podocytopathies [9]. It is now recognized that the common feature in minimal change disease (MCD), membranous glomerulopathy, crescentic glomerulonephritis, collapsing glomerulopathy, focal segmental glomerulosclerosis (FSGS), diabetic nephropathy, and lupus nephritis is through podocyte damage and dysfunction [10]. Early events in the damaged podocyte are alterations of the slit diaphragm, reorganization of the foot process structure with the fusion of filtration slits, and apical displacement. These preliminary changes may not be visible under an optical microscope, necessitating the use of electron microscopy [10]. Early podocyte structural change is characterized by detachment of podocytes from the glomerular basement membrane. These changes can lead to severe and progressive glomerular injuries, if the condition persists. Hence, early recognition of any podocyte injury is of clinical importance.

## Methods

This review is intended to explore aspects associated with the use of Nephrin as a marker in early podocyopathies and it is not the intention of the authors to comment on the molecular function of the protein nephrin. We performed a review of the literature with PubMed, US National Library of Medicine, EMBASE, and The Cochrane Database of Systematic Reviews to determine whether urinary nephrin could be used to detect early glomerular injury. We used the following keywords: nephrinuria, urinary nephrin, microalbuminuria, podocytes, and glomerular injuries. The keywords were searched alone or in combination with other keywords. We reviewed articles published between January 1990 and July 2014. Our search identified a total of 19 studies (6 in animals; 13 in human) that have been published to date. The most common clinical condition in which urinary nephrin analyses were carried out were diabetic nephropathy, glomerulonepharitis, pre-eclampsia, and Systemic Lupus Erythematosus (SLE).

### Nephrin measurement

The most commonly used method for urinary nephrin measurement was with the commercially available ELISA kit ((Exocell, Philadelphia, PA; R&D Systems, Minneapolis, MN, USA) [11–16]. In one study, the amount of podocyturia was also measured using urinary flow cytometry [17]. Other methods included messenger ribonucleic acid real-time polymerase chain reaction (mRNA RT-PCR) [18–20], electrophoresis [21–25]. In one study, the nature of the analysis was not described [26]. Neprin measurement using a commercially available ELISA kit has increasingly becoming the method of choice for studies conducted since 2012. However, no studies have compared the efficacy of various methods for measuring urinary nephrin. In a few studies, other podocyte protein measurements (Podocalyxin [14] and Podocin [22]) were also carried out concurrently with urinary nephrin but there were no attempts to compare the sensitivity and specificity of these urinary markers to each other.

### Animal studies

Disease models investigated by animal studies can be divided in 2 groups – nephrosis and diabetic nephropathy. In both models, urine nephrin was detected early, prior to significant albuminuria. Luimula et al. carried out a study in which experimental glomerular disease was induced in rats by using puromycin aminonucleoside (PAN) [21]. PAN nephrosis results in effacement and fusion of the podocyte foot processes, leading to the loss of the ultrafiltration barrier, closely resembling the functional and morphologic changes of minimal change disease. The investigators detected nephrin in urine in the peak proteinuric samples and concluded that urinary nephrin is an important marker of proteinuric diseases. The investigators were however unable to determine nephrin’s role in this study. In another study [22], the investigators used Heymann nephritis (a widely used experimental model to study idiopathic membranous nephropathy) [27] to induce glomerular injury and to investigate the excretion of urinary nephrin as a result of this injury. The investigators showed that nephrin was excreted into urine during the initial stages of Heymann nephritis, prior to any abnormal albuminuria. However, the investigators from this study did not recognize this nephrinuria as an early sign of glomerular injury and concluded that nephrinuria may have contributed to the development of proteinuria.

Aaltonen et al. investigated the role of nephrin in the pathogenesis of diabetic nephropathy [23]. In this study, the investigators used both the classic streptozotocin(STZ) model of rat diabetes (selectively impairing the insulin production in pancreatic beta cells, mimicking type I diabetes) and spontaneously diabetic non-obese diabetic mice (a model for human insulin-dependent diabetes mellitus). The investigators found that nephrin was detectable in urine of STZ-induced rats at 4 weeks, which then peaked by 6 weeks. Urinary nephrin was detected before the peak in albuminuria and the investigators concluded that nephrin was related to the early changes of diabetic nephropathy. However, again the authors concludedthat the appearance of nephrin in urine contributed to the loss of glomerular function and failed to recognise this as a marker of podocyte injury. In a study by Alter et al., a group of 23 Wistar rats were uninephrectomized and randomly assigned to two groups [28]. The study group received streptozotocin (STZ) to induce diabetes mellitus, whereas the control group received citrate buffer without STZ. Blood glucose levels, blood pressure, kidney function, and weight were then measured. After 18 weeks, blood and urine specimens were collected, and then the animals were euthanized and their organs harvested. The animals in the diabetic group were found to have an elevated glucose level with normal systolic blood pressure. The investigators found that while there were no significant differences in renal function and urinary albumin level between the two groups, the urinary excretion of nephrin was significantly higher in the diabetic group. Nephrin appeared even before albuminuria, leading the study’s authors to conclude that nephrin measurement in urine offers the possibility for early detection of nephropathy. In another study, Chang et al. demonstrated similar findings in Akita mice (a mouse model of type 1 diabetes mellitus resulting from spontaneous point mutation in the *Ins2* gene) [11]. In this study, the investigators found that urinary nephrin excretion was significantly increased in albuminuria in study of 16 and 20 week old mice and that this correlated with the urinary albumin excretion rate. Enhanced urinary nephrin excretion was shown to be associated with kidney injury and was detected early in the disease process. A similar finding was also reported by O’Brien *et al* in another study [12]. Table 1 summarizes the animal studies that have been published to date.

**Table 1:** **Summary of the animal studies that have investigated the relationship between nephrinuria and glomerular injury**

Authors	Journal (Year)/PMID	Study subjects (sample size N)	Disease model	Method of urine	Results
				Nephrin assay	
Luimula et al.	Pediatric Research (2000)/11102543	Sprague–Dawley rats	Puromycin aminoglycoside (PAN) induced nephrosis (Minimal change disease) Effacement and fusion of	Electrophoresis	1. Animals treated with puromycin developed albuminuria starting at day 3, and reached maximum at day 6
		Control = 6 Study = 12			2. Nephrin can be detected among urinary proteins when the level of proteinuria exceeds 25 mg/mL
Nakatsue et al.	Kidney Int. (2005)/15882266	Wistar rats	Idiopathic membranous nephropathy (Heymann nephritis)	Electrophoresis (Western blot)	1. Nephrin is excreted into urine in the early stages of nephritis
		Control = 5 Study = 16			
		Sprague–Dawley rats Control = 3 Study = 3			
Aaltonen et al.	Lab. Invest (2001)/11555666	Wistar rats	Type 1 (Insulin-dependent) diabetes mellitus and Diabetic nephropathy	Electrophoresis	1. Free nephrin was found in the urine of STZ-induced rats at 4 weeks and was at the maximum at 6 weeks
		Control = 3 Study = 14			
		Non Obese diabetic mice			2. Nephrin is connected to the early changes of diabetic nephropathy
		Control = 3 Study = 9			
Alter et al.	Clin.Lab (2012)/22997966	Wistar rats	Streptozotocin (STZ) induced Diabetes Mellitus and diabetic nephropathy	ELISA (USCN Life Sci. Inc. Burlington, NC, USA)	1. Nephrinuria detected prior to albuminuria and renal impairment.
		Control = 9 Study = 14			2. This biomarker offer an advantage to urinary albumin with respect to early detection
Chang et al.	Plos One (2012)/22496773	Akita mice	Type 1 diabetes mellitus and diabetic nephropathy	ELISA (Exocell, Philadelphia, PA)	1. Urinary nephrin excretion is associated with kidney injury and is detectable early in the disease process.
		Control = 8 Study = 8			2. Onset of hyperglycemia associated with nephrinuria
					3. Nephrinuria rate has a positive correlation with albuminuria
O’Brien et al.	J of Diabetic Research (2013)/Not available	Mice	Obesity, Type 2 diabetes	ELISA (Exocell, Philadelphia, PA)	1. Nephrinuria correlates with albuminuria
		Control = 12 Study = 12			

### Human studies

Urinary nephrin measurements were carried out in 3 clinical conditions: diabetic nephropathy, nephrosis (including Lupus nephritis) and preeclampsia.

Compared with the animal studies, human studies involved larger sample size but on some occasions there were no control groups [24].

Wang *et al* carried out a study to investigate the gene expression of podocyte-associated molecules in the urinary sediment of 21 patients with diabetic nephropathy and 9 healthy controls [18]. The investigators found that urinary nephrin levels (measured by mRNA expression) correlated with proteinuria (r = 0.502, p = 0.020) but not with eGFR. Nephrinuria was greater in diabetics with nephropathy, and the investigators concluded that nephrin measurement could play a role in clinical stratification of the patients’ diabetic nephropathy. Do Nascimento et al. used mRNA RT-PCR to measure urine nephrin in a cohort of 15 controls and 67 diabetics [19]. The study subjects were assigned to 3 different groups: normoalbuminuria (NO) (<30 mg/g creatinine); microalbuminuria (MI) (30–300 mg/g creatinine); and macroalbuminuria (MA) (>300 mg/g creatinine). The investigators found that urine nephrin was higher in diabetics than in non diabetics and correlated with increasing albuminuria. Nephrinuria was found in 53%, 71%, and 90% of NO, MI and MA diabetes subjects, respectively (p = 0.023). The investigators concluded that diabetic subjects had elevated urinary mRNA levels of podocytes protein such as nephrin compared to non- diabetic subjects, even the NO patients. In another study, Patari et al. demonstrated the presence of nephrin fragments using immunohistochemistry and western blotting techniques in the urine of type 1 diabetics with or without nephropathy [25]. The study consisted of five cohorts of patients: 1) diabetic patients with normal albuminuria; 2) diabetics with microalbuminuria; 3) diabetics who had developed microalbuminuria recently; 4) diabetics with macroalbuminuria; and 5) a control group consisting of healthy adults. Micro-albuminuria was defined as a 24-h urinary albumin excretion rate (AER) of 30–300 mg in two of three consecutive 24-h urine collections; macroalbuminuria as an AER of 300 mg/24 h; and normoalbuminuria as a persistent AER of 30 mg/24 h. The investigators found that nephrinuria was present in 30% of normoalbuminuric, 17% of microalbuminuric, 28% of macroalbuminuric, and 28% of new microalbuminuric patients; however, none of the control subjects were nephrinuric. The authors concluded that the glomerular filtration barrier may already be compromised in one-third of diabetic patients with nephrinuria.

In a recent study, Jim et al. investigated whether the detection of nephrin in urine can be used as an early biomarker of diabetic nephropathy [13]. Firstly, renal histopathology in a group of 15 patients with type 2 diabetes was compared for the protein expression of nephrin with a group of 12 control patients. This investigation showed the statistically significant down-regulation of podocin and nephrin in kidney biopsies for diabetic nephropathy. Further, analysis of the study group, based on the amount of albumin in urine, detected nephrinuria in 100% of diabetic patients with micro- and macroalbuminuria and in 54% of patients with normoalbuminuria. Nephrinuria correlated with albuminuria (rho = 0.89, p = 0.001) and systolic blood pressure (rho = 0.32, p = 0.007). It also correlated negatively with serum albumin (rho = 20.48, p = 0.0001) and eGFR (rho = 20.33, p = 0.005). The investigators concluded that nephrinuria is a reliable biomarker of preclinical diabetic nephropathy.

In another study, Ng et al. investigated the association between nephrinuria and various renal traits [24]. This study, which recruited 381 patients with type 2 diabetes mellitus, involved urinary nephrin analysis using electrophoresis and Western blot analysis. The electrophoretic pattern revealed that nephrin was excreted in four distinct protein bands (25, 50, 60 and 75 kDa) with the 60 kDa molecule being the most common presentation (40.7%). By using regression analysis, the investigators showed that each nephrin fragment was associated with a decrease in eGFR and that nephrinuria was strongly associated with the urine albumin/creatinine ratio. This finding was similar to that of another study involving 70 patients with diabetes mellitus [26].

Proletov et al. investigated daily urinary excretion of nephrin as a possible marker for podocyte apoptosis [29]. In this study, 71 patients with biopsy proven primary glomerulonephritis were recruited. The investigators found that urinary nephrin excretion (using ELISA) correlated well with daily level of proteinuria (r = 0.67, p < 0.05). Proteinuria and nephrinuria levels were lower in patients undergoing treatment with cyclosporine. The study showed that the more severe the degree of podocyte injury (as evidenced by increased urinary nephrin), the worse the level of proteinuria. Response to treatment is evidenced by not only a decrease in urinary protein levels, but also nephrin excretion. In another very similar study, urine nephrin levels in a cohort of 74 adults with chronic glomerulonephritis were measured, and the investigators found that urine nephrin level exhibited a positive correlation with the severity of the disease and proteinuria [17]. Urine nephrin measurement has also been used in studies involving patients with lupus nephritis (LN) [20]. In this study, a group of 49 (32 with active LN and 17 in remission) patients with LN were recruited. mRNA was used determine urine nephrin levels, and the researchers found that it was elevated in the active group and showed a positive correlation with proteinuria (r0.480, p < 0.01). The authors concluded that urinary nephrin quantification could play a role in the clinical classification of patients with LN.

More recently, urinary nephrin analyses have been used to monitor pre-eclampsia and pregnancy-induced hypertension. Wang et al. investigated the role of podocyte protein shedding in pre-eclampsia, recruiting 34 pregnant women and dividing them into three groups (normotensive, chronic hypertension, and pre-eclampsia) [14]. Clinical information such as weight, blood pressure, and gestation was recorded, and urine creatinine, albumin, and nephrin were analyzed. The investigators found that nephrin was barely detectable in the urine specimens of normal pregnant women and of those that had chronic hypertension. In pre-eclamptic patients, however, urinary nephrin concentrations were significantly increased. These results provide strong evidence that podocyte protein shedding occurs in pre-eclampsia at levels that correlate with proteinuria. In another study, Son et al. compared urine excretion in a cohort of pregnant women with severe pre-eclampsia compared with normotensive pregnant women [30]. The investigators found urinary nephrin to be significantly higher in women with severe pre-eclampsia and a positive correlation between urinary nephrin and urine protein level. Urine nephrin concentrations also correlated with diastolic blood pressure and serum creatinine levels in the severe pre-eclamptic group. The investigators concluded that urinary nephrin excretion plays a critical role in the pathogenesis of proteinuria during pre-eclampsia and that it is a good indicator of renal damage. A similar conclusion was also made in another study by Mehta et al. of 67 high-risk obstetric patients [15]. In a more recent study, Jim et al. found the sensitivity and specificity for nephrinuria in detecting pre-eclampsia to be only 57% and 58%, respectively [16]. Table 2 summarizes human studies on nephrin measurement published to date.

**Table 2:** **Summary of the human studies**

Authors	Journal (Year)/PMID	Study sample size	Clinical condition	Method of urine	Results
				Nephrin assay	
Patari et al.	Diabetes (2003)/14633858	Control = 29	Diabetic Nephropathy	Electrophoresis (Western blot)	1. Nephrinuria was present in 30% of normoalbuminuric, 17% of microalbuminuric, 28% of macroalbuminuric,
		Study = 120			2. None of the control subjects was nephrinuric.
Wang et al.	Nephron Clin Pract. (2007)/17596726	Control = 9	Diabetic nephropathy	mRNA RT-PCR	1. Nephrinuria higher in patients with diabetic nephropathy compared to control.
		Study = 21			2. Nephrinuria correlated with proteinuria
Ng et al.	Nephrol. Dial.Transplant (2011)/21196468	Study = 381	Diabetic nephropathy	Electrophoresis (Western blot)	1. Each Nephrin fragment was associated with a decline in eGFR
					2. Nephrinuria was strongly associated with the urine albumin/creatinine ratio
					3. Nephrinuria was significantly associated with lower eGFR even among normoalbuminuric patients (ACR ≤30 mg/g)
Jim et al.	PLoS ONE (2012)/22615747	Control = 12	Diabetic nephropathy	ELISA (Exocell, Philadelphia, PA)	1. Nephrinuria preceded microalbumniuria
		Study = 15			2. Nephrinuria detected in all diabetic patients with micro and macroalbuminuria
					3. Nephrinuria also correlated significantly with albuminuria and systolic blood pressure
					4. Correlated negatively with serum albumin and eGFR
do Nascimento et al.	BMC Nephrol.(2013)/24103534	Control = 15	Diabetic Nephropathy	mRNA RT-PCR	1. Normoalbuniuric diabetics showed increased urinary nephrin compared to control group
		Study = 67			2. Nephrinuria correlated with diabetic nephropathy stages
Petrica et al.	*Nephrology Dialy.Transpl (2014)/NA	Control = 11	Diabetic nephropathy	NA	1. Urine albumin creatinine ratio correlates with nephrinuria
		Study = 70			2. Increased levels of urinary nephrin in normoalbuminuric and microalbuniuruc patients
Wang et al.	J Rheumatol (2007)/17985404	Control = 17	Systemic Lupus Errythematosus (SLE)	mRNA RT-PCR	1. Nephrinuria higher in patients SLE nephritis
		Study = 32			2. Nephrinuria correlated with proteinuria
					3. Nephrinuria level correlated with severity of SLE disease activity
Tchebotareva et al.	*Nephrology Dialy.Transpl (2012)/NA	Study = 74	Glomerulonephritis	ELISA	1. Nephrinuria correlated with proteinuria and severity of GN
					2. Immunosuppressive therapy lowers nephrinuria levels
Proletov et al.	*Nephrology Dialy.Transpl (2014)/NA	Study = 71	Glomerulonephritis	ELISA	1. Nephrinuria correlated with daily proteinuria
					2. Nephrinuria and proteinuria lowest excretion in patients treated with Cyclosporine.
Mehta et al.	*AJKD (2012)/NA	Control = 14	Preeclampsia	ELISA (Exocell, Philadelphia, PA)	1. Urine nephrin to creatinine ratio (UNCR) are predictive of preeclampsia in second trimester of pregnancy
		Study = 67			
Wang et al.	Am J Physiol Renal Physiol (2012)/22301621	Control = 8	Preeclampsia	ELISA (Exocell, Philadelphia, PA)	1. Urinary Nephrin and podocalyxin concentrations were significantly increased and highly correlated with each other in preeclampsia
		Study = 26			2. Nephrin and podocalyxin were also correlated with urine protein concentrations in preeclampsia
					3. Nephrin undetectable in normal pregnancy
Son et al.	Eur J Obstet Gynecol Reprod Biol. (2013)/23116596	Control = 30	Preeclampsia	ELISA (R&D Systems, Minneapolis, MN, USA)	1. Nephrin was significantly higher in women with severe pre-eclampsia
		Study = 43			2. Positive correlation between urinary nephrin and urine protein level
					3. Urine nephrin concentrations correlated with diastolic blood pressure and serum creatinine levels in the severe pre-eclamptic group
Jim et al.	PLoS ONE (2014)/25010746	Control = 13	Preeclampsia	ELISA (Exocell, Philadelphia, PA)	1. Alb/Cr ratio had a high specificity (96%). All three biomarkers exhibited poor positive predictive values (14–62%) but acceptable negative predictive values (89–91%).
		Study = 78			

**mRNA RT-PCR**: Messenger Ribonucleic acid Real-time Polymerase Chain Reaction; ACR = Albumin creatinine ratio; *- Conference abstract.

## Conclusion

Currently, urine micro-albuminuria is used as an early indicator of glomerular injury. Both animal and human studies have demonstrated that nephrinuria occurs early in glomerular injury, preceding albuminuria, and that there is a positive correlation with severity of renal diseases. Neprin detection in urine may also have role in early detection of severe pre-eclampsia. Urine nephrin analysis thus has the potential to become an important biomarker of early glomerular injury. To date, no studies on children or adolescents have been published, pointing to a need for clinical studies using urinary nephrin to assess, monitor, and prognosticate renal diseases in children.
